# Determination of a serum total calcium concentration threshold for accurate prediction of ionized hypercalcemia in dogs with and without hyperphosphatemia

**DOI:** 10.1111/jvim.15654

**Published:** 2019-11-06

**Authors:** Elizabeth M. Groth, Dennis J. Chew, Jody P. Lulich, Megan Tommet, Aaron K. Rendahl, Brian D. Husbands, Eva Furrow

**Affiliations:** ^1^ Department of Veterinary Clinical Sciences, College of Veterinary Medicine University of Minnesota St. Paul Minnesota; ^2^ Department of Veterinary Clinical Sciences, College of Veterinary Medicine The Ohio State University Columbus Ohio; ^3^ Department of Veterinary and Biomedical Sciences University of Minnesota St. Paul Minnesota

**Keywords:** calcium, canine, hypercalcemia, ionized calcium, phosphorus

## Abstract

**Background:**

Total serum calcium (tCa) concentrations are poorly predictive of ionized calcium (iCa) status in dogs.

**Hypothesis:**

There is an optimal threshold of tCa concentration that is highly predictive of ionized hypercalcemia and this threshold is higher in hyperphosphatemic dogs as compared to nonhyperphosphatemic dogs.

**Animals:**

Nonhyperphosphatemic (n = 1593) and hyperphosphatemic (n = 250) adult dogs.

**Methods:**

Retrospective medical record review of paired tCa and iCa concentration measurements in dogs presented to a university teaching hospital over a 5‐year period. Positive and negative predictive values, sensitivity, and specificity were calculated for tCa concentration thresholds of 11.0‐15.0 mg/dL (upper limit of laboratory reference interval = 11.5 mg/dL) in nonhyperphosphatemic and hyperphosphatemic groups.

**Results:**

In nonhyperphosphatemic dogs, an optimal tCa concentration threshold of 12.0 mg/dL resulted in a positive predictive value of 93% (95% confidence interval [CI], 84%‐98%) and sensitivity of 52% (95% CI, 43%‐61%) for ionized hypercalcemia. An optimal tCa concentration threshold was not identified for hyperphosphatemic dogs. The nonhyperphosphatemic dogs had a higher prevalence of ionized hypercalcemia than the hyperphosphatemic dogs (7 versus 3%, *P* = .04) and a lower prevalence of ionized hypocalcemia (23 versus 62%, respectively; *P* < .001).

**Conclusions and Clinical Importance:**

High tCa concentrations are strongly predictive of ionized hypercalcemia in nonhyperphosphatemic adult dogs and should prompt further diagnostic testing to determine the underlying cause of hypercalcemia. In this population, dogs without increased tCa concentrations rarely had ionized hypercalcemia, but iCa concentrations still should be evaluated in patients with tCa concentrations within the reference interval if there is clinical suspicion for calcium abnormalities.

**Abbreviations**
iCa: ionized calcium
NPV: negative predictive value
PPV: positive predictive value
tCa: total calcium

## 1 INTRODUCTION

Hypercalcemia in dogs often is an indicator of serious disease with the most common cause being malignant neoplasia such as lymphoma or anal gland adenocarcinoma.^1^ Less common causes of hypercalcemia include hypoadrenocorticism, primary hyperparathyroidism, and hypervitaminosis D.^2^ Persistent hypercalcemia, regardless of underlying cause, can have serious consequences including soft‐tissue mineralization and subsequent renal failure. The serious nature of such diseases and complications requires an accurate way to detect hypercalcemia so that a diagnosis can be made and appropriate intervention can be instituted as soon as possible. Ionized calcium (iCa) is the biologically active form and is the most relevant to clinical practice.^2^ However, iCa concentration measurements are not readily available in all practice settings, and serum total calcium (tCa) concentration often is used in its place which is of questionable acceptability.

A large study in dogs showed significant diagnostic discordance between tCa or adjusted (corrected) calcium and iCa status. This study evaluated a single tCa concentration threshold for defining hypercalcemia (>12 mg/dL), based on the upper limit of the laboratory reference interval.^3^ The positive predictive value (PPV) at this threshold was relatively low at 68%. Dogs with ionized hypercalcemia can have tCa concentrations that are normal to very high, but tCa concentration medians have been reported to be well above reference intervals.^4^ This suggests that there may be an optimal cutoff for tCa concentration that can more reliably predict hypercalcemia in dogs.

When exploring the optimal diagnostic threshold for tCa concentration, its utility for predicting ionized hypercalcemia historically has been evaluated separately in subpopulations of patients with and without azotemia. Previous reports have shown that tCa concentration was less predictive in dogs with chronic kidney disease as compared to dogs that were nonazotemic.^5^ In fact, in dogs with kidney disease, using the upper limit of the laboratory reference interval as the tCa concentration threshold resulted in a PPV of only 27% for predicting ionized hypercalcemia.^3^ Azotemia affects the diagnostic discordance between tCa and iCa concentrations because of increased complexed calcium, with phosphorus as the most commonly measured complexed ion.^6^ Citrate and lactate also are important ions that complex with calcium, but they are difficult to measure in a clinical setting. Evaluation of the discordance of tCa concentration to predict iCa concentration has not been separately evaluated in light of the presence hyperphosphatemia, despite the high frequency of hyperphosphatemia in azotemic dog populations.^7^ Therefore, it is important to reinvestigate the use of tCa concentrations in populations categorized as nonhyperphosphatemic or hyperphosphatemic.

Our objective was to evaluate the accuracy of tCa concentrations for predicting ionized hypercalcemia across multiple tCa concentrations thresholds in dogs with and without hyperphosphatemia. Our hypothesis was that there is a threshold for tCa concentration which is strongly predictive of ionized hypercalcemia. We predicted that this threshold would be higher in patients with hyperphosphatemia compared to those without.

## 2 MATERIALS AND METHODS

### 2.1 Case selection

Medical records from the University of Minnesota, Veterinary Medical Center between 2011 and 2016 were reviewed for patients with measurements of both of serum tCa and blood iCa collected within 12 hours of each another. Information obtained from the medical records included age, breed, sex, time between iCa and tCa determination, as well as phosphorus, BUN, and creatinine concentration. A final diagnosis was recorded for all patients with ionized hypercalcemia. Dogs <1 year of age were considered pediatric and were not included in the primary analyses because the reference interval used for iCa concentration was determined based on adult dogs. Adult dogs were divided into 2 groups based on the presence of hyperphosphatemia as defined by a serum phosphorus concentration above the upper limit of the laboratory reference interval (3.3‐6.8 mg/dL). Dogs also were classified as azotemic or not, defined either as BUN concentration >31 mg/dL or serum creatinine concentration > 1.6 mg/dL based on the upper limits of the laboratory reference intervals. For concentrations that were above or below the limit of detection, the reported results were rounded to the detection limit (ie, if BUN was reported as >240 mg/dL, it was rounded to 240 mg/dL). If multiple pairs of calcium measurements were obtained on an individual dog during a single visit, only the pair with the shortest time between sample collections was included in the analysis. Samples from the same dog were included in the analysis if the samples were obtained during different hospital visits (discharged in between). All iCa measurements were obtained using a hand‐held analyzer (iSTAT 1, Abbott Point of Care Inc, East Windsor, New Jersey). Venous samples were collected in a dry lithium heparin syringe. The iCa concentration reference range was determined in‐house using 63 healthy dogs aged 2‐8 years old and resulting in a reference interval of 5.1‐5.9 mg/dL. Serum tCa, phosphorus, BUN, and creatinine concentrations were measured using standard reference laboratory methods (AU480 Chemistry analyzer, Beckman Coulter, Inc, Brea, California); the canine reference interval for tCa concentration was 9.3‐11.5 mg/dL.

### 2.2 Statistical analysis

Thresholds of tCa concentration were selected in 0.5 mg/dL increments between 11.0 and 15.0 mg/dL. Sensitivity, specificity, PPV, and negative predictive value (NPV) were calculated at each of these thresholds for the nonhyperphosphatemic and hyperphosphatemic groups; 95% confidence intervals (CIs) were calculated using the Clopper‐Pearson exact method. To determine an optimal tCa concentration threshold for predicting hypercalcemia, the PPVs were evaluated to identify the lowest threshold (and therefore highest sensitivity) with a PPV >90%. Comparison of proportions of ionized hyper‐, hypo‐, and normocalcemia between the nonhyperphosphatemic and hyperphosphatemic groups were performed using a Cochran Armitage trend test, followed by Chi‐squared tests to evaluate each status individually. A Chi‐squared test also was used to compare the proportions of azotemic dogs between the hyperphosphatemic and nonhyperphosphatemic groups. A Pearson's correlation was performed to determine the relationship between tCa and iCa concentrations for both the nonhyperphosphatemic and hyperphosphatemic groups. Wilcoxon rank‐sum tests were used to compare age, and iCa, tCa, BUN, and creatinine concentrations between nonhyperphosphatemic and hyperphosphatemic groups and to compare iCa concentrations between the true positives and false negatives. All calculations were carried out using R software for statistical computing (R, version 3.3.1. http://www.r-project.org).

## 3 RESULTS

A total of 2334 medical records were reviewed. Four‐hundred and ninety‐one visits were excluded from analysis: 404 were excluded because of multiple samples for an individual dog within the same hospital visit, 80 were excluded because the dog was pediatric (<1 year old), and 7 were excluded because of incomplete medical records. The remaining 1843 visits from 1688 adult dogs were included for analysis. The nonhyperphosphatemic group comprised 1593 visits and the hyperphosphatemic group comprised 250 visits. A total of 1617 purebred and 71 mixed breed dogs were included in the study. The purebred population comprised 134 breeds; the most common breeds represented included Labrador Retriever (n = 227), Golden Retriever (n = 104), German Shepherd Dog (n = 74), Dachshund (n = 70), and Yorkshire Terrier (n = 61). The median time between the tCa and iCa concentration measurements was 43 minutes (range, 0 minutes to 12 hours).

Data including age, tCa concentrations, BUN, serum creatinine, serum phosphorus concentrations, and prevalence of ionized hyper‐, hypo‐, and normocalcemia for the nonhyperphosphatemic and hyperphosphatemic groups are presented in Table 1. The nonhyperphosphatemic group was younger than the hyperphosphatemic group (*P* < .001). Azotemia was more common in the hyperphosphatemic group than in the nonhyperphosphatemic group (91 versus 19%, respectively; *P* < .001); the median BUN and serum creatinine concentrations are shown in Table 1. The iCa concentration was significantly higher in the nonhyperphosphatemic group as compared to the hyperphosphatemic group (*P* < .001). The prevalence of ionized hypocalcemia was significantly higher in the hyperphosphatemic group than in the nonhyperphosphatemic group, whereas ionized hypercalcemia and normocalcemia were more common in the nonhyperphosphatemic group.

**Table 1:** Population characteristics for 1842 paired ionized and total calcium concentration measurements from dogs. Continuous variables are reported as median (range)

Variable	Nonhyperphosphatemic (n = 1593)	Hyperphosphatemic (n = 250)	*P* value
Age, years	8.6 (1.0‐17.5)	9.8 (1.3‐18.5)	**<.001**
tCa, mg/dL	9.7 (3.1‐19.3)	9.5 (4.4‐14.7)	.48
iCa, mg/dL	5.3 (2.3‐9.7)	4.9 (1.8‐7.3)	**<.001**
BUN	17 (3‐140)	76 (5‐240)	**<.001**
Creatinine	.9 (.2‐7.5)	2.9 (.3‐21.3)	**<.001**
Phosphorus	4.0 (1.0‐6.8)	9.4 (6.9‐28.3)	ND^a^
iCa status^b^			**<.001**
Hypercalcemic	7% (n = 109)	3% (n = 8)	**.04**
Hypocalcemic	23% (n = 370)	62% (n = 154)	**<.001**
Normocalcemic	70% (n = 1114)	35% (n = 88)	**<.001**

*Note*: *P* values in bold denote significance (<.05).

Abbreviations: iCa, ionized calcium; ND, not determined; tCa, total calcium.

a Serum phosphorous concentration was not statistically compared between nonhyperphosphatemic and phosphatemic groups, as this criteria was used to define the groups.

b A Cochran Armitage trend test was used to determine if iCa status (proportion of dogs with hypocalcemia, normocalcemia, and hypercalcemia) differed between nonhyperphosphatemic and hyperphosphatemic groups, followed by Chi‐squared test to compare proportions individually.

**Figure 1:**
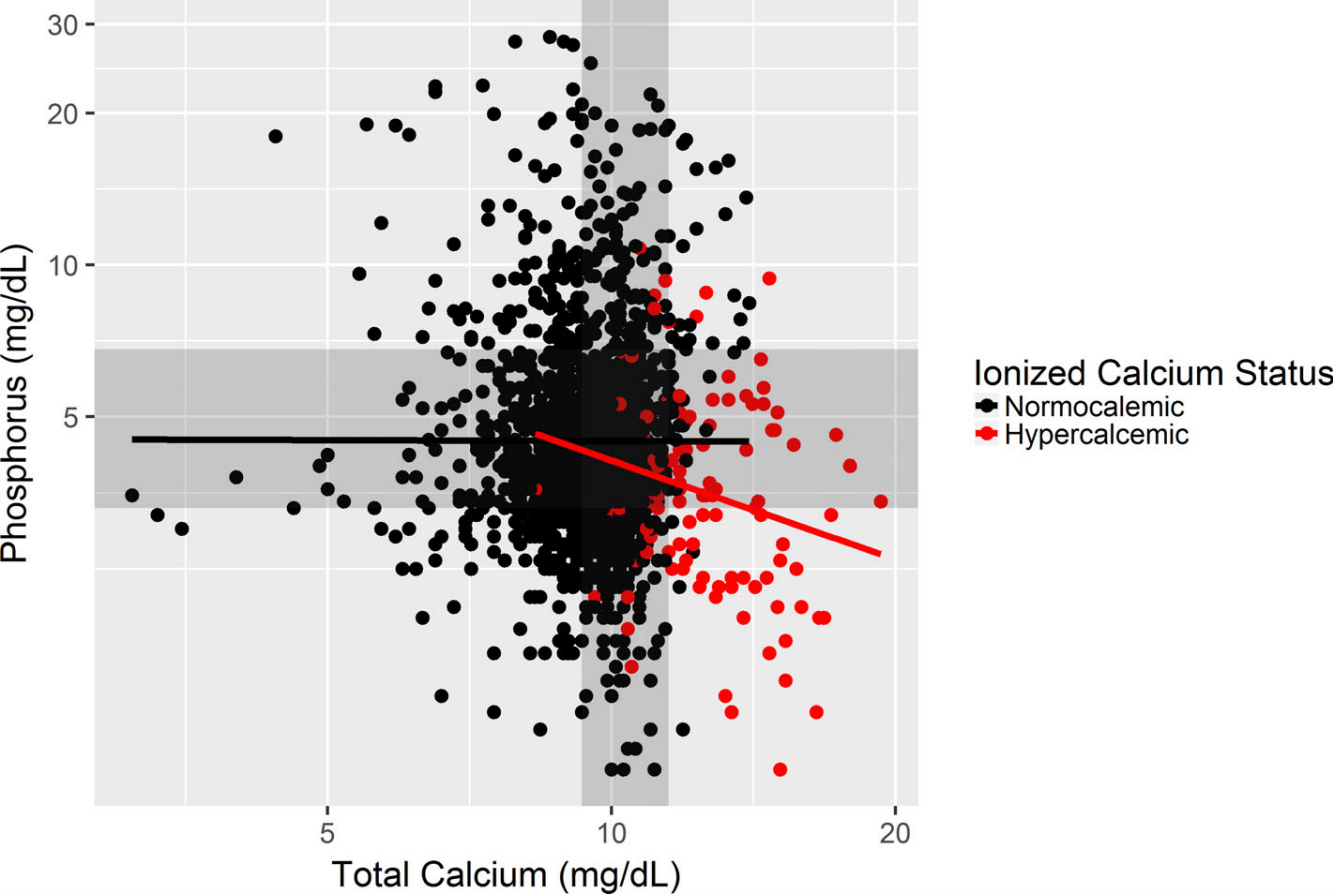
Scatter plot of phosphorus and total calcium concentrations. Each dot represents an individual dog. The x‐ and y‐scales are log‐transformed to more accurately depict the correlations between these variables, as they were not normally distributed. In dogs with ionized hypercalcemia (red dots and line), there was a weak negative correlation (*r* = −.23, 95% CI, −.39 to −.05; *P* = .01) between phosphorus and total calcium concentrations. No correlation was present in nonhypercalcemic dogs (black dots and line; *r* = 0, 95% CI, −.05 to .05; *P* = .94)

The final diagnoses of all dogs with ionized hypercalcemia are presented in Table 2; the most common diagnoses were hypercalcemia of malignancy (53/112, 47%) and primary hyperparathyroidism (25/112, 22%). In dogs with ionized hypercalcemia, there was a weak negative correlation between tCa and phosphorus concentrations (*r* = −.23; 95% CI, −.39 to −.05; *P* = .01); no correlation was present in dogs without hypercalcemia (Figure 1). The tCa concentration was significantly correlated with iCa concentration in both the nonhyperphosphatemic and hyperphosphatemic groups (*r* = .75; 95% CI, 0.67‐0.72; *P* < .001 and *r* = .67; 95% CI, 0.59‐0.73; *P* < .001, respectively).

**Table 2:** Final diagnosis and serum total calcium status of 113 dogs (duplicates removed) with ionized hypercalcemia

	# Elevated tCa/# elevated iCa (proportion)		
Diagnosis, number	Nonhyperphosphatemic	Hyperphosphatemic^a^	All^a^
Malignancy	35/49 (.71)	2/4 (.50)	37/53 (.70)
Lymphoma	19/21 (.90)	1/1 (1.00)	20/22 (.91)
ASA	7/10 (.70)	NA	7/10 (.70)
Other carcinoma	4/5 (.80)	NA	4/5 (.80)
Plasma cell	0/1 (0)	0/1 (0)	0/2 (0)
Insulinoma	0/1 (0)	NA	0/1 (0)
Sarcoma	2/4 (.50)	0/1 (0)	2/5 (.40)
Uncharacterized	2/7 (.29)	1/1 (1.00)	3/8 (.38)
PHPTH	22/25 (.88)	NA	22/25 (.88)
Renal	0/1 (0)	0/3 (0)	0/4 (0)
CKD	NA	0/2 (0)	0/2 (0)
AKI	NA	0/1 (0)	0/1 (0)
CKD and AKI	0/1 (0)	NA	0/1 (0)
Hypoadrenocorticism	2/5 (.40)	NA	2/5 (.4)
Granulomatous	0/4 (0)	NA	0/4 (0)
Xylitol toxicity	0/1 (0)	NA	0/1 (0)
Calcitriol treatment	NA	1/1 (1.00)	1/1 (1.00)
Open	8/19 (.42)	0/1 (0)	8/20 (.40)
Total	65/104 (.63)	3/8 (.38)	68/112 (.61)

Abbreviations: ASA, anal sac adenocarcinoma; iCa, ionized calcium; PHPTH, primary hyperparathyroidism; tCa, total calcium.

a One hyperphosphatemic dog had hemangiosarcoma and chronic kidney disease. Therefore, the totals for the hyperphosphatemic group and full population are one less than the sum of the individual counts for the diagnosis categories.

**Figure 2:**
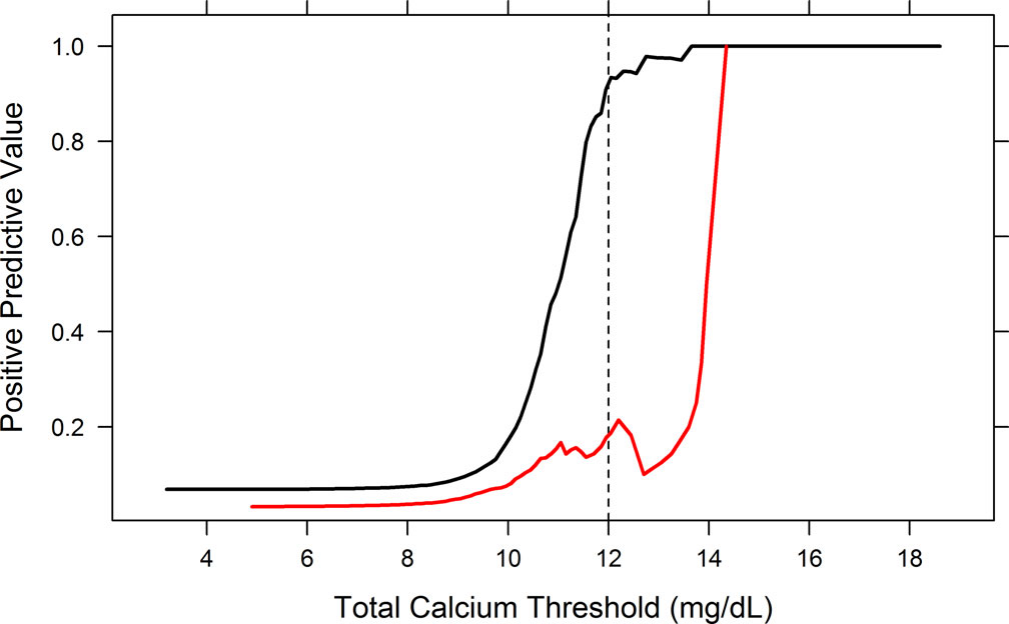
Positive predictive value (PPV) of total calcium concentration for the prediction of ionized hypercalcemia in nonhyperphosphatemic (black line) and hyperphosphatemic (red line) adult dogs. The vertical dotted lines represent the designated optimal total calcium threshold for non‐ hyperphosphatemic dogs (12 mg/dL). No predictive threshold was identified for hyperphosphatemic dogs

**Figure 3:**
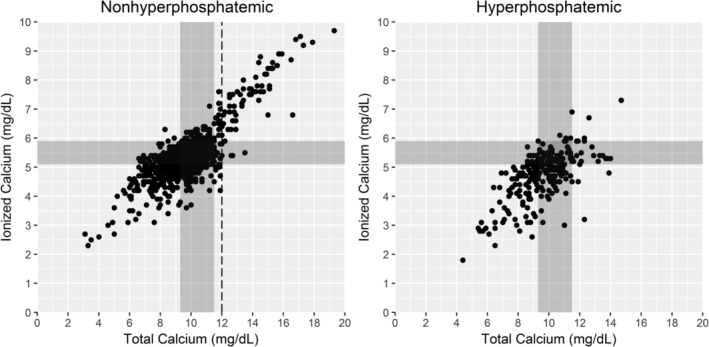
Scatter plot of ionized and total calcium concentrations in nonhyperphosphatemic and hyperphosphatemic adult dogs. Each dot represents an individual dog. The shaded area on each plot represents the values within the reference intervals of ionized and total calcium concentrations. The dotted line represents the proposed threshold for using total calcium concentration to predict ionized hypercalcemia nonhyperphosphatemic dogs

The PPV increased rapidly for tCa concentrations thresholds >10 mg/dL in the nonhyperphosphatemic group. The PPV for the hyperphosphatemic group increased steeply at 14 mg/dL, but only a single dog fell above that threshold (Figure 2). The sensitivity, specificity, PPV, and NPV at each of the evaluated thresholds are presented in Table 3 (nonhyperphosphatemic group) and Table 4 (hyperphosphatemic group). For the nonhyperphosphatemic group, the optimal tCa concentration threshold for predicting ionized hypercalcemia was 12.0 mg/dL with a PPV of 93% (95% CI, 84%‐98%). The NPV for this threshold was 97%, and the sensitivity and specificity were 52 and 100%, respectively. An optimal threshold was not determined for the hyperphosphatemic group because of low PPV, sensitivity, or both at all thresholds evaluated. Figure 3 shows data from individual dogs in relation to tCa, iCa, and the optimal threshold of the nonhyperphosphatemic group.

**Table 3:** PPV, NPV, sensitivity, and specificity data for thresholds of total calcium concentration used to predict ionized hypercalcemia in 1593 nonhyperphosphatemic dogs. The prevalence of ionized hypercalcemia in this population was 7%. The optimal total calcium threshold for prediction in this population is represented in bold

Total Calcium Threshold	PPV (95% CI), #true positive/#test positive	NPV, #true negative/#test negative	Sensitivity, #true positive/#with disease	Specificity, #true negative/#without disease
11	.51 (.43‐.59), 80/156	.98 (.97‐.99), 1408/1437	.73 (.64‐.81), 80/109	.95 (.94‐.96), 1408/1484
11.5	.80 (.70‐.88), 71/89	.97 (.97‐.98), 1466/1504	.65 (.55‐.74), 71/109	.99 (.98‐.99), 1466/1484
**12**	**.93 (.84‐.98), 57/61**	**.97 (.96‐.97), 1480/1532**	**.52 (.43‐.61), 57/109**	**1.00 (.99‐1.00)**, 1480/1484
12.5	.94 (.84‐.99), 49/52	.96 (.95‐.97), 1481/1541	.45 (.35‐.55), 49/109	1.00 (.99‐1.00), 1481/1484
13	.98 (.87‐1.00), 39/40	.95 (.94‐.96), 1483/1553	.36 (.27‐.46), 39/109	1.00 (1.00‐1.00), 1483/1484
13.5	1.00 (.89‐1.00), 33/33	.95 (.94‐.96), 1484/1560	.30 (.22‐.40), 33/109	1.00 (1.00‐1.00), 1484/1484
14	1.00 (.88‐1.00), 29/29	.95 (.94‐.96), 1484/1564	.27 (.19‐.36), 29/109	1.00 (1.00‐1.00), 1484/1484
14.5	1.00 (.84‐1.00), 21/21	.94 (.93‐.95), 1484/1572	.19 (.12‐.28), 21/109	1.00 (1.00‐1.00), 1484/1484
15	1.00 (.78‐1.00), 15/15	.94 (.93‐.95), 1484/1578	.14 (.08‐.22), 15/109	1.00 (1.00‐1.00), 1484/1484

Abbreviations: iCa, ionized calcium; NPV, negative predictive value; PPV, positive predictive value; tCa, total calcium.

**Table 4:** PPV, NPV, sensitivity, and specificity data for a total calcium concentration of 12 mg/dL to predict ionized hypercalcemia in 250 hyperphosphatemic dogs. The prevalence of ionized hypercalcemia in this population was 3%. No optimal total calcium threshold was identified, as all cut offs had a low PPV, low sensitivity, or both

Total Calcium Threshold	PPV (95% CI), #true positive/#test positive	NPV, #true negative/#test negative	Sensitivity, #true positive/#with disease	Specificity, #true negative/#without disease
11	.17 (.07‐.31), 7/42	1.00 (.97‐1.00), 207/208	.88 (.47‐1.00), 7/8	.86 (.80‐.90), 207/242
11.5	.14 (.03‐.35), 3/22	.98 (.95‐1.00), 223/228	.38 (.09‐.76), 3/8	.92 (.88‐.95), 223/242
12	.19 (.04‐.46), 3/16	.98 (.95‐.99), 229/234	.38 (.09‐.76), 3/8	.95 (.91‐.97), 229/242
12.5	.18 (.02‐.52), 2/11	.97 (.95‐.99), 233/239	.25 (.03‐.65), 2/8	.96 (.93‐.98), 233/242
13	.13 (0‐.53), 1/8	.97 (.94‐.99), 235/242	.13 (0‐.53), 1/8	.97 (.94‐.99), 235/242
13.5	.20 (0.01‐.72), 1/5	.97 (.94‐.99), 238/245	.13 (0‐.53), 1/8	.98 (.96‐1.00), 238/242
14	1.00 (.03‐1.00), 1/1	.97 (.94‐.99), 242/249	.13 (0‐.53), 1/8	1.00 (.98‐1.00), 242/242
14.5	1.00 (.03‐1.00), 1/1	.97 (.94‐.99), 242/249	.13 (0–.53), 1/8	1.00 (.98‐1.00), 242/242
15	NA, 0/0	.97 (.94–.99), 242/250	0 (0–.37), 0/8	1.00 (.98‐1.00), 242/242

Abbreviations: iCa, ionized calcium; NPV, negative predictive value; PPV, positive predictive value; tCa, total calcium.

For the nonhyperphosphatemic group, we further evaluated the performance of the tCa concentration threshold of 12 mg/dL in dogs with (n = 302) and without azotemia (n = 1291). Data are presented in Table 5. The sensitivity of tCa concentration for predicting ionized hypercalcemia was lower in the nonazotemic group (44%) than in the azotemic group (74%; *P* = .008). The specificity was higher in the nonazotemic group (*P* = .02) but was excellent for both groups (≥99%). The PPV and NPV were not significantly different between groups. We further evaluated the population of nonhyperphosphatemic dogs by comparing iCa concentrations between true positives versus false negatives. Dogs with false negatives had a significantly lower median iCa concentration (6.1 mg/dL; range, 6.0‐7.1 mg/dL) than those with true positives (7.5 mg/dL; range, 6.1‐9.7 mg/dL; *P* < .001).

**Table 5:** PPV, NPV, sensitivity, and specificity data for thresholds of total calcium concentration used to predict ionized hypercalcemia in 1593 nonhyperphosphatemic dogs separated into 2 groups based on the presence (302 dogs) or absence (1291 dogs) of azotemia. Performance was compared between the azotemic and nonazotemic groups with Chi‐square tests

Group	PPV (95% CI), #true positive/#test positive	NPV, #true negative/#test negative	Sensitivity, #true positive/#with disease	Specificity, #true negative/#without disease
Nonazotemic dogs	.97 (.85‐1.00), 34/35	.96 (.95‐.97), 1212/1256	.44 (.32‐.55), 34/78	1.00 (.99‐1.00), 1212/1213
Azotemic dogs	.88 (.70‐.98), 23/26	.97 (.94‐.99), 268/276	.74 (.55‐.88), 23/31	.99 (.97‐1.00), 268/271
*P* value	.41	.75	**.008**	**.02**

*Note*: *P* values in bold denote significance (<.05).

## 4 DISCUSSION

In our study population, a tCa concentration >12.0 mg/dL was highly predictive of ionized hypercalcemia in adult dogs without hyperphosphatemia. Because of the low prevalence of ionized hypercalcemia (6%), the NPV was high at all thresholds. However, the sensitivity (proportion of patients with disease that are correctly classified as positive) of tCa concentration for detecting ionized hypercalcemia was low at 52%. Thus, if there is clinical suspicion for hypercalcemia (such as malignancy or ectopic mineralization), an iCa concentration should be evaluated in the face of a normal tCa concentration.

The PPV was prioritized when determining the optimal tCa concentration threshold rather than sensitivity. The PPV is the probability that a patient with a positive test result has the condition in this case the probability a dog with an increased tCa concentration has ionized hypercalcemia. The PPV and NPV are the most relevant statistics for a clinical scenario when an individual patient with unknown status is being screened.^8^, ^9^ In contrast, sensitivity and specificity are useful when comparing the performance of a test to a reference standard but provide little practical application to an individual patient with unknown disease status. The disadvantage of PPV is that it is affected by disease prevalence. When a disease is common, the PPV will increase, but a low prevalence will decrease the PPV. It is also important to note that a high PPV is not always necessary. A moderate threshold can be considered when the risk, convenience, and expense of follow‐up screening are low.^8^, ^9^ We determined all 4 major metrics (PPV, NPV, sensitivity, and specificity) for multiple thresholds of tCa concentration in our study so that clinicians can make their own decisions about how to apply the results in their practice.

We were unable to determine an optimal tCa concentration threshold for predicting ionized hypercalcemia in hyperphosphatemic dogs. Phosphorus is 1 of the commonly measured anions that complexes with calcium. Other anions such as citrate and lactate also could contribute to complexed calcium, but these analytes were not measured. Complexes of calcium can increase the tCa concentration independent of changes in iCa concentration. In these instances, tCa concentration can be increased above the reference interval, but iCa concentration can be low, normal, or high. Hyperphosphatemia is common in certain disease processes such as advanced chronic kidney disease. Ionized hypocalcemia is more common than ionized hypercalcemia in these instances because of decreased calcitriol synthesis and the complexing effect of hyperphosphatemia on iCa.^3^, ^10^

The landmark study on predicting iCa status from tCa concentration in dogs concluded that it is not acceptable to use tCa concentration to predict iCa concentration in dogs.^3^ The tCa concentration threshold used in that study was identical to the optimal threshold identified in this study, 12.0 mg/dL, and yet, we conclude that tCa concentration can be an excellent predictor of iCa status. The reason for the opposing conclusions stems from 2 considerations in the application of tCa concentration for predicting iCa status: (1) whether it is being used to predict hypercalcemia or hypocalcemia and (2) whether the dog has a condition expected to cause discordance between tCa and iCa concentrations, namely azotemia or hyperphosphatemia. In the previous study, the PPV for 12.0 mg/dL was 68% for prediction of ionized hypercalcemia and 77% for prediction of ionized hypocalcemia in the overall study population. However, when performance was tested on dogs without chronic kidney disease, PPV increased to 90% for ionized hypercalcemia; PPV for ionized hypocalcemia remained low at 74%. Our study classified dogs based on phosphorus status rather than kidney disease, and we found that azotemia does not significantly decrease the PPV of tCa concentration for prediction of ionized hypercalcemia in nonhyperphosphatemic dogs.

A recent study in cats with and without azotemic chronic kidney disease showed similar performance of tCa concentration for predicting ionized hypercalcemia as in the present study.^11^ The study of cats found moderate correlation between tCa and iCa concentrations in both the nonazotemic and azotemic groups (*r* = .6 for both). They reported that tCa concentration above the reference range (11.8 mg/dL) was very specific for ionized hypercalcemia in both nonazotemic and azotemic cats (100 and 99.9%, respectively), but sensitivity was low at 9% in the nonazotemic group and 28% in the azotemic group. When the tCa calcium threshold was lowered to 10.5 mg/dL, which was below the upper limit of the laboratory reference range, it remained highly specific in the nonazotemic group (90%) with much improved sensitivity (95%). A lower tCa concentration threshold of 10.3 mg/dL also improved sensitivity in the azotemic group (72%) but decreased the specificity (71%). However, although these thresholds optimize the combination of sensitivity and specificity, they do not take into account the effects on PPV. Although not directly reported, PPV can be calculated from the data. The 10.5 mg/dL threshold had a PPV of 56%, and the 10.3 mg/dL threshold in the azotemic group had a PPV of 31%. These tCa concentration thresholds therefore have low accuracy for the prediction of ionized hypercalcemia, because half or more of the tCa concentration at or above the thresholds are false positives. This study in cats did not find a significant effect of serum phosphorous concentration on the risk of hypercalcemia. However, most cats included in the study had normal serum phosphorus concentrations.

In a clinical setting, mild increases in tCa concentration may be attributed to biologic variation, especially in clinically normal dogs, owing to its high index of individuality.^12^ However, our current study shows that increases in tCa concentration are strongly predictive of ionized hypercalcemia. In our study population, the PPV was 93% for a tCa concentration >12.0 mg/dL and 80% for a tCa concentrarion >11.5 mg/dL (the upper limit of the laboratory reference range). Thus, even mild increases in tCa concentration in a nonhyperphosphatemic adult dog should prompt further diagnostic investigation including confirmation of iCa concentration status to determine if it is concordant or not. Several neoplastic, as well as non‐neoplastic, diseases can cause hypercalcemia, and early recognition of these diseases could lead to earlier intervention and potentially increased duration of life. Ionized hypercalcemia was rare in dogs without abnormally high tCa concentrations (NPV of 97% for the threshold of 12.0 mg/dL and 98% for the threshold of 11.5 mg/dL). However, it is important to recognize the effect that disease prevalence will have on NPV. In patients with clinical suspicion for hypercalcemia, the prevalence of true hypercalcemia could be much higher than in our study population, resulting in a lower NPV and thus more false negatives if tCa concentration is used to detect ionized hypercalcemia. This is particularly true in cases with mild increases in iCa concentration. Therefore, we again emphasize the need to measure an iCa concentration in patients with clinical suspicion for hypercalcemia even if the tCa concentration is within the reference range.

Another important finding from our study is that an increase in tCa concentration is a poor predictor of ionized hypercalcemia in hyperphosphatemic dogs (PPV = 19% for tCa concentration > 12 mg/dL and 15% for tCa concentration > 11.5 mg/dL). Many hyperphosphatemic dogs in our study had mild increases in tCa concentration without concurrent increases in iCa concentration, and an optimal threshold for tCa concentration could not be determined in the hyperphosphatemic group. All thresholds evaluated either had low PPV, low sensitivity, or both. The NPV was ≥97% for all thresholds, indicating again that ionized hypercalcemia is rare in dogs without increases in tCa concentration, but, as discussed above, this only applies to populations expected to have a similarly low prevalence of ionized hypercalcemia. One of the explanations for the lower proportion of hyperphosphatemic dogs with ionized hypercalcemia is the effect of the most common causes of ionized hypercalcemia (malignancy and primary hyperparathyroidism) on decreasing serum phosphorus concentration. As shown in Figure 3, there was a negative correlation between tCa and serum phosphorus concentrations in dogs with ionized hypercalcemia, and hypophosphatemia was common.

Findings from our study may allow expanded analysis in future retrospective research studies. The poor correlation between tCa and iCa historically has made it difficult to interpret calcium status in studies where only tCa concentration was reported. Using the upper limit of the laboratory reference interval for tCa concentration has a reasonably high PPV for nonhyperphosphatemic dogs. In contrast, >80% of hyperphosphatemic dogs with an increased tCa concentrations do not have ionized hypercalcemia. Failure to account for the poor performance of tCa concentration regardless of the serum phosphorous concentration could confound the ability to detect the impact of hypercalcemia on clinical conditions of interest. For example, a study on mediastinal lymphoma found no association between the presence of hypercalcemia and survival.^13^ However, more than half of the dogs classified as hypercalcemic had only tCa concentration data available, and although serum phosphorus concentration was not specifically reported, the authors stated that azotemia was common (present in 48% of hypercalcemic dogs).

Classifying hypercalcemia based on tCa concentration is common practice in veterinary oncology. Several recent studies have presented conflicting information regarding the relationship between the presence of hypercalcemia, prognosis, and tumor type. Hypercalcemia is commonly associated with T‐cell lymphoma which has a negative prognosis factor as compared to other types of lymphoma. However, other tumor types are more variable. Anal gland tumors are reported to cause hypercalcemia in 16%‐53% of cases.^14^, ^15^, ^16^, ^17^ One study found the presence of hypercalcemia to be a poor prognostic indicator.^14^ However, other studies have failed to demonstrate this prognostic association.^15^, ^16^, ^17^ Similar conflicting information also is published with respect to multiple myeloma.^18^, ^19^ It is possible that some of the discrepancies among these studies could be secondary to type 2 error (failing to detect a difference when 1 truly exists) because of misclassification of calcium status. We propose that when iCa concentration is not available (eg, retrospective studies), tCa concentration can be used as a surrogate to classify calcium status in nonhyperphosphatemic adult dogs but it is not sufficiently accurate for dogs with hyperphosphatemia.

A recent study developed a model for predicting iCa status in dogs based on numerous results available on a biochemical profile in dogs.^20^ This model had high PPV (90%) and specificity (99.6%), and moderate sensitivity (64%). Using this model can be cumbersome because 6‐10 variables from routine serum biochemistry are entered into a complex equation which is used to estimate iCa concentration. Additionally, the use of this model has not been validated with variables measured from multiple laboratories with different reference intervals, which would be critical before application in a clinical setting because models often perform worse on external validation.^21^ In our study, the optimal tCa concentration threshold in nonhyperphosphatemic dogs had similar performance to the aforementioned multivariable model with a PPV of 93%, specificity of 100%, and sensitivity of 52%. This suggests that a simple approach using only the tCa concentration is sufficient, as long as the dog is not hyperphosphatemic.

Our study had some limitations inherent to its retrospective design. Although most tCa and iCa concentration measurements were performed in <1 hour of each other, we allowed for up to 12 hours between measurements. Treatments that patients received during this time period that may have altered either measurement were not recorded. Two other limitations pertain to the application of results in different populations of dogs. As discussed above, we selected tCa concentration thresholds based on PPV rather than sensitivity and specificity, which is disease prevalence dependent. Therefore, the PPV for the threshold designated in our study may be different in other patient populations. All of the cases included in our study either were hospitalized patients or those referred for specialty evaluation, and the prevalence of hypercalcemia could be different in patients presented for outpatient evaluation or evaluation in a general practice setting. Finally, reference ranges are calculated based on specific methodology and are laboratory specific. The optimal tCa concentration threshold for prediction of ionized hypercalcemia could differ in other laboratories using other chemistry analyzers. Of note, the previous study in dogs used a different chemistry analyzer, but also obtained a high PPV (90%) for a tCa concentration threshold of 12.0 mg/dL in dogs that did not have chronic kidney disease.^3^

In conclusion, we showed that even mildly increased tCa concentration (> 12.0 mg/dL) are highly predictive of ionized hypercalcemia in adult dogs without hyperphosphatemia. There is no optimal threshold for patients with hyperphosphatemia. Ionized hypercalcemia is rare in dogs without increases in tCa concentration, regardless of phosphorous status. This information has important case management and research implications. Additional studies are needed to further validate these thresholds using other reference laboratories and patient populations.

## CONFLICT OF INTEREST DECLARATION

Authors declare no conflict of interest.

## OFF‐LABEL ANTIMICROBIAL DECLARATION

Authors declare no off‐label use of antimicrobials.

## INSTITUTIONAL ANIMAL CARE AND USE COMMITTEE (IACUC) OR OTHER APPROVAL DECLARATION

Authors declare no IACUC or other approval was needed.

## HUMAN ETHICS APPROVAL DECLARATION

Authors declare human ethics approval was not needed for this study.
